# Structured chronic primary care and health-related quality of life in chronic heart failure

**DOI:** 10.1186/1472-6963-9-104

**Published:** 2009-06-19

**Authors:** Marije Bosch, Trudy van der Weijden, Richard Grol, Henk Schers, Reinier Akkermans, Louis Niessen, Michel Wensing

**Affiliations:** 1 Scientific Institute for Quality of Healthcare, Radboud University Nijmegen Medical Centre, Nijmegen, The Netherlands; 2 Scientific Institute for Quality of Healthcare/Department of General Practice, School for Public Health and Primary Care (Caphri), Maastricht University, Maastricht, The Netherlands; 3 Department of General Practice, Radboud University Nijmegen Medical Centre, Nijmegen, The Netherlands; 4 Department of International Health, John Hopkins Bloomberg School of Public Health, Baltimore, USA; 5 School of Medicine, Policy and Practice, University of East Anglia, Norwich, UK

## Abstract

**Background:**

Structured care is proposed as a lever for improving care for patients with chronic conditions. The purpose of this study was to explore the associations of structured care characteristics, derived from the Chronic Care Model, with health-related quality of life (HRQOL) and optimal clinical management in chronic heart failure (CHF) patients in primary care, as well as the association between optimal management and HRQOL.

**Methods:**

Cross-sectional observational study using multi-level random-coefficient analyses of a representative sample of 357 patients diagnosed with CHF from 42 primary care practices in the Netherlands. We combined individual medical record data with patient and physician questionnaires.

**Results:**

There was large variation in the levels and presence of structured care elements. A 91% of physicians indicated that next appointments for CHF patients were made immediately after visits, while 11% indicated that reminders on CHF management were periodically received in their practice. Few associations were found between the organizational characteristics and optimal treatment or HRQOL. Optimal pharmacological treatment related to better quality of life (β = -11.5, *P *< .0001). Also, more lifestyle advice was given in practices with an appointment system allowing contact with more than one professional during the encounter (β = 1.0, *P *= .04).

**Conclusion:**

HRQOL and treatment quality in CHF patients were not consistently associated with characteristics of structured care in primary care practices.

## Background

In high-income countries, the prevalence of chronic heart failure (CHF) is estimated to be 1–2% [1], and expectations are that this figure will be rising as survival of acute heart disease is increasing [1,2]. CHF has high hospital admission rates [3], and severely compromises health-related quality of life (HRQOL) [4]. Many CHF patients are managed in primary care, but research on organization of primary care for CHF is limited. Guidelines on the management of CHF recommend pharmacological treatment to deal with heart failure symptoms and to reduce morbidity and mortality in nearly all patients: in particular ACE inhibitors (ACEI) or angiotensin-II receptor blockers (ARB) and β-blockers [5-7]. In addition, clinical guidelines [5-7] incorporate principles of structured chronic care such as patient counselling on self management to promote continuity of care. Patients are to be monitored regularly with (daily) body weight measurements and are to receive lifestyle advice, such as reducing salt intake, limiting fluid intake, exercise, and resting periods.

The goal of guidelines is to improve the survival and quality of life of patients in daily life. Consistently, studies show suboptimal adherence to guidelines in management of heart failure, especially in primary care [8,9]. There is a growing belief that structural support at the organizational level is needed to enhance guideline implementation [10-12]. Structured chronic care is considered increasingly important to optimize clinical management of patients with chronic diseases [13,14]. Studies in diabetes indicated that the level of the organizations' use of physician reminders [15], performance feedback [15,16], involvement of patients in defining treatment goals [16], patient education [17] and structured care management [15,16] is positively associated with better outcomes. There is evidence that improvements in health care delivery improve health outcomes as HRQOL and limit the need for hospitalizations as well as improve prescribing practices for patients with chronic heart failure [13,18-22]. However, little is known about the relationship between the presence of these elements and indicators of quality of CHF care in primary care. Such insight is important, since it may guide the design of future practice models for heart failure care to improve HRQOL in CHF patients.

In this study we explored the extent to which structured chronic care features, derived from the Chronic Care Model, are associated with HRQOL and optimal management in heart failure in primary care. We also studied the associations between clinical management and HRQOL.

## Methods

### Design and population

In the period 2005–2006, we performed an observational study including 72 GPs in 42 primary care practices in the Netherlands. The sample of practices accounted for urbanization rate and types of practice.

GPs received tailored written instructions how to extract a list of patients with CHF from their electronic medical record system (EMR). Subsequently, they were asked to assess whether the patients on the EMR list met the diagnostic criteria of the ICPC code K77 (heart failure). This was to limit the number of false-positive CHF diagnoses. All patients with a diagnosis of CHF *according to the GP *were eligible to be included. Reasons for exclusion were: terminal illness, Dutch language problems, mental impairment, or other practical reasons to not include the patient in this study. GPs sent their patients (893 in total) an invitational letter, asking for informed consent. Consent was received from 511 patients. Ethical approval for this study was waived by the ethics committee Arnhem-Nijmegen.

### Outcome measures

HRQOL was measured through the Dutch version of the Minnesota Living with Heart Failure Questionnaire [23]. This disease-specific 21-item questionnaire has been widely used in clinical trials and shows documented reliability, validity and sensitivity [23,24]. The instrument uses a six-point Likert scale, indicating to what extent CHF influences patient's life during the month previous to measurement (ranging from 0: no influence, to 5: HF influenced the patient's life to a very high extent). If an item was not applicable to a patient, a '0' was recorded. Per patient, a total score was computed by summing the 21 items (summary range 0–105), with the lower scores reflecting better HRQOL. We imputed missing values (per person mean substitution). The scale ratings were excluded if less than 16 answers per patient were entered. Internal consistency reliability, using Chronbach's alpha was 0.94.

Our second outcome measure was the sum score on eight measures of lifestyle advice to CHF patients [5-7] (see Table 1; measurements at individual patient level). Here, Chronbach's alpha was 0.86. A patient receives a score between 0 and 8, as each indicator received either a score of '1' or '0'. All patient items were assessed through printed questionnaires that were sent to 461 patients around the date of medical record data collection. All patients that handed in a written informed consent received a questionnaire, unless the patient had died in the meantime, or there were reasons for exclusion as judged by the GP. After three weeks reminders were sent. Questionnaires were received from 385 patients (83.5% response rate).

**Table 1:** characteristics of the patients (N = 357)

Age (mean years, SD)	75.7 (10.2)
Sex (% male)	52.9
NYHA class (% I & II)	73.1
Optimal pharmacological treatment (% yes)	33.1
ACE/ARB (%)	58.3
β-blockers	46.9
Spironolactone	31.0
Lifestyle advice (0 – 8) (mean, SD)	4.4 (2.7)
Heart signs and symptoms (% yes)	58.3
Type of heart disorder	52.8
Medication intake	54.2
Reduced salt and limited fluid intake	37.5
Physical activity	41.6
Flu prevention	91.1
Weighing regularly	54.7
Coping behaviour	42.8
Quality of Life (0 – 105, less is better) (mean, SD)	30.5 (24.8)
Men	26.3 (1.9)
Women	35.5 (2.4)
NYHA class I	15.5 (1.5)
NYHA class II	36.6 (2.4)
NYHA class III	51.5 (2.7)
NYHA class IV	57.4 (11.3)
≤ 75	27.0 (2.3)
> 75	33.5 (2.0)

Our third outcome measure was a dichotomous variable that indicated whether a patient had received optimal pharmacological treatment. This variable was based on a global adherence index (GAI) for key-pharmacological recommendations [5-7]. For each patient, this index indicated the proportion of evidence-based recommendations followed by the GP out of the total number of recommendations that applied for that particular patient [25]. The GAI included the prescription of ACEI (or ARB) for all patients, β-blockers of proven efficacy in CHF (bisoprolol, carvedilol or metoprolol) [5-7] in patients with previous MI or NYHA class ≥ II) and spironolactone (in patients NYHA class ≥ III). The measure was scored '1' for each individual patient if the patient received all indicated drugs. Information on pharmacological treatment was obtained from scrutinizing the patients' records by trained research staff. Due to limited resources, collection of medical record data was limited to a random sample of a maximum of 15 patients per practice.

### Independent measures

Organizational characteristics were measured using written validated questionnaires for physicians sent to all 72 GPs in our sample. They worked in 49 physician groups in 42 separate practices (response rate 88%). We distinguished between general organizational characteristics such as location of organization, list size, and age and number of years of experience of physicians and aspects of structured chronic care including items addressing four of the domains of the Chronic Care Model [13,14,26]: self-management support, design of the care delivery system, decision support, and supportive clinical information system (Table 2). Self-management support emphasizes patients' responsibility in managing their health through such strategies as resolving problems, and devising action plans. An active delivery system design facilitates planned patient visits and includes the existence of practice teams with a division of tasks. Decision support enhances adherence to evidence-based guidelines, incorporated in daily practice decision making through system reminders and/or prompts. They are reinforced through provider training or other decision support mechanisms. Clinical information systems provide access to patient data and can be used to plan individual patient's care, identify relevant subpopulations for care, and monitor the performance of health care providers. Items are based on the elements as specified in the Assessment of Chronic Illness Care instrument [27], which was translated from English to Dutch by a bilingual researcher, followed by back-translation from Dutch to English by a second bilingual researcher. Discrepancies between the original questionnaire items and the back-translation were identified and solved with a third bilingual researcher. All researchers were familiar with the theoretical constructs. Items were either dichotomous (e.g. "Is there someone responsible for self-management in patients with CHF in your practice?"), or based on a five-point Likert scale ranging from 'always' to 'never' (e.g. "Are CHF patients involved in making treatment plans?"), which were rescaled to a binary variable ('never' and 'rarely' = 0; 'regularly', 'usually', and 'always' = 1). Items were excluded if they were missing in >10% of cases. The remaining items are listed in Table 2. Since the internal consistency as measured by Chronbach's alpha of the scales varied widely (ranging from 0.27 to 0.75), we used the single items in the analyses. In addition, we calculated a sum score of the structured care characteristics, ranging from 0 to 17.

**Table 2:** structured care characteristics in 49 physician groups

**Characteristic**	**(% yes)**
Regular clinical meetings on CHF patients	18.8
Special hours for patients with heart disease	10.4
Agreements with cardiologist on sharing of information and organization of care	24.5
*Decision support*	
• Info materials present for patients regarding guideline adherence	58.5
• Presence of HF protocol in practice	88.6
*Delivery system design*	
• Next appointment made immediately after visit	91.1
• Continuity of care for CHF patients is a high priority	88.9
• Frequency and content of visit are tailored to individual patients	100.0
• Clear tasks and responsibilities practice members	31.8
• Someone who assures that tasks and responsibilities are clearly defined	34.9
• Appointment system facilitates the patient seeing multiple practice employees in a single visit	**13.8 #**
*Self-management support*	
• Assessment and documentation of self-management needs and activities is part of the treatment	84.4
• Patient involvement in treatment plans	86.7
• Someone responsible for self-management in patients with HF	16.3
*Clinical information systems*	
• Reminders build in EMR	25.0
• Reminders are periodically received	11.1
• Information related to the needs of HF patients is provided to practice members	16.7
*Sum score structured care characteristics (0–17; mean, SD)*	8.3 (2.7)

# significant association with lifestyle advice: β = 1.0; 95% CI (0.0, 2.0); p = 0.04

### Data-analysis

Patients without a date of diagnosis or medical record data were excluded from the study (N = 121). Thirty three (6.4%) patients had died between inclusion and medical record abstraction. In total, 154 patients were excluded, leaving 357 patients for this study. Excluded patients did not differ significantly from included patients with respect to age, sex, and NYHA class.

We analyzed data on the patient level. Patient data was merged with physician data. In case several GPs were seeing the same patients their data were aggregated before merging. In case of continuous variables, means were calculated across physicians within the same physician group (one or more physicians seeing the same panel of patients). If disagreements existed between physicians regarding the dichotomous variables, such as presence of a HF protocol in practice, they were contacted for clarification. In a few remaining cases, scores higher then '0', were scored '1'. The aggregated GP data were then merged with the patient data set, in such a way that each patient treated by more than one physician had the same value on these particular GP variables. Descriptive analyses of patients' characteristics were performed (Table 1). For the description of physician characteristics, means and proportions were calculated across physician practice groups.

Bivariate associations were explored between the structured care characteristics on the one hand, and management and HRQOL on the other, using random-coefficient regression analyses for the HRQOL and the lifestyle advice outcomes, and random-coefficient logistic regression analyses for the dichotomous outcome (optimal pharmacological treatment). If items showed hardly any variation between physicians (<10%), they were excluded from the bivariate analyses. Random-coefficient analyses were performed to correct for the clustering effect of the design, patients (level 1) were clustered within physician groups (level 2). For statistically significant associations (*P *< .05), we repeated the analyses using patient age, gender and – in case of the lifestyle management and HRQOL outcomes – NYHA class as possible confounders. In addition, we explored whether optimal pharmacological management and lifestyle advice were related to HRQOL in random-coefficient regression analyses using the same confounders. All analyses were performed using SPSS 14, except for the multi-level logistic regression analyses that were performed using the Glimmix procedure in SAS for Windows V8.2.

## Results

### Patient characteristics

Table 1 shows the characteristics of the patients. 73% of patients were classified as NYHA I or II. The mean age of the patients was 75.7 years (SD 10.2), and 52.9% was male. Around 33% of patients received optimal pharmacological treatment, the average number of lifestyle advice patients received was 4.4 (SD 2.7). The mean HRQOL score was 30.5. Hence, on average, patients scored 1.45 on the Likert scales (ranging from 0: no influence, to 5: CHF influenced the patient's life to a very high extent). As expected, scores varied by NYHA class; the higher the NYHA class, the lower the reported HRQOL scores were (*P *< .001). Patients above 75 years of age reported lower HRQOL as compared to patients younger than 75 (*P *= .02).

### Characteristics of primary care physicians

About 63% of the physician groups were (small) group practices. The mean age of the GPs across groups was 49.2 years and mean number of years of experience since qualification as a GP was 18.6.

Table 2 presents the various physician groups as they structured their care for CHF patients. The mean sum score for the structured care characteristics was 8.3 (SD 2.7). Few physician groups (10%) had special hours for heart disease patients, while around 19% held regular clinical meetings on CHF. Practice nurses and assistants were involved in care for cardiovascular risk patients to a high extent; in 81% they were involved in systematically determining risk profiles; in 98% they did regular check-ups of known patients with CHF, and in 56% they were involved in case-finding. Finally, in 94% of physician groups they provided patients with oral or written information. A 75% of the physician groups had written agreements on when assistants and nurses should ask for feedback from their GP. A 10% of the groups used standardized forms for referral to specialized care.

### Associations between structured care characteristics and HRQOL

No associations were found between the factors presented in Table 2 and HRQOL. Bivariate analyses showed that in practices in which someone was responsible for self-management in patients with CHF, patients reported better quality of life (β = -9.91, *P *= .03). Also, in practices in which materials were provided to practice members regarding the needs of CHF patients, patients reported better HRQOL (β = -9.71, *P *= .03). However, both associations were not statistically significant when we repeated the analysis adjusting for patient age, sex and NYHA class (β = -3.6, *P *= .27, and β = -3.8, *P *= .21 respectively).

### Associations between clinical treatment and HRQOL

Optimal lifestyle advice was not related to HRQOL, whereas optimal pharmacological treatment did relate to HRQOL. Patients who received optimal pharmacological treatment reported better HRQOL (β = -11.5, *P *< .0001). Adding the control variables did not change this relation.

### Associations between structured care characteristics and optimal treatment

No associations were found between the organizational factors and optimal pharmacological treatment. In bivariate analyses, it appeared that more lifestyle advice was given in practices with an appointment system allowing contact with more than one professional during the encounter (β = 1.0, *P *= .04). Patients scored one point higher on the sum score for lifestyle advice (scale from 0 to 8) compared with patients in practices not allowing for appointments with several care givers within one visit. Adding our control variables did not change this relationship.

## Discussion

Contrary to the expectation, HRQOL and treatment in chronic heart failure patients were not consistently associated with aspects of structured care characteristics of primary care practices. Our study involved a representative sample of general practices in the Netherlands. At the time of the study, no specific arrangements with insurers existed that may have influenced treatment.

Earlier studies that focused on the relationship between 'structured care principles' and quality of (primary) care for various other conditions showed mixed results. One study that examined the chronic care model in preventing health risk behaviours in primary care found some associations e.g. between point-of-care reminders, clinical staff meetings and recommended services [20]. Also, a study in primary diabetes care found that planned care – the implementation of practice guidelines, support for self-management and clinical information systems – was associated with improved performance and most metabolic outcomes in patients on 2-year follow-up [28]. In addition, a study on the relation between 8 measures of primary health care orientation and the implementation of 11 elements of chronic care management in 957 US physician organizations found that 6 of their 8 measures, including health education activity, were positively associated with adoption of chronic care elements [29]. However, a study that tested whether improvements in care quality were correlated with changes in the chronic care model in 17 primary care clinics concluded that despite implementation of the chronic care model and improvements in quality measures for three chronic conditions, there were very few significant relations between these changes [30]. Yet, their diabetes control measures were significantly associated with both clinical information systems and decision support. In addition, a cross-sectional study on the association between quality of care and intensity of three disease management strategies (provider feedback, reminders, and structured care) found that more intense disease management strategies predicted higher scores on many process of care measures, but only one intermediate outcome and one medication management outcome [15].

So, although not conclusive, the studies show some associations between structured care and improved management of conditions, whereas our study mostly failed to show associations between structured care principles and clinical management. Possibly, this may be explained by variation in outcome measures. The amount of variation in our outcome measures that could maximally be attributed to cluster level factors – as measured by the intra-cluster correlation (ICC) – was for all three study outcomes smaller than 10% (ranging from 1.5 to 8.2%). The variation in outcomes at practice level may have been higher in other studies. Also, both the observational studies had somewhat larger sample sizes than our study [211, 306]. However, a post hoc sample size calculation for multiple regression analysis revealed that our sample size gave us 80% power to detect a R^2 ^of 0.049 (for both HRQOL and lifestyle advice), assuming a type one error rate of 5% and a maximum of 4 factors in the model. This is comparable with a effect size for multiple regression f^2 ^of 0.051, which is a small effect according to Cohen [31,32].

Systematic reviews [19,21,33] on the effectiveness of comprehensive disease management programs (though mainly based on secondary care studies) in improving clinical outcomes in HF, indicated that chronic care elements may improve both HF management as well as QOL. For instance, whereas we failed to find associations between structured care elements and improved pharmacological management, two of the 3 trials McAlister et al. included in their review that assessed the medications of proven efficacy, demonstrated greater use of these therapies in the intervention studies. However, these improvements on prescribing do not necessarily seem to be translated into improvements of QOL. Studies investigating the relation between pharmacological management and QOL mostly did not find an association [34,35]. The fact that we did find a significant relationship between optimal pharmacological treatment and HRQOL may be explained by our measure of optimal pharmacological treatment. This measure indicates whether the patients received all the indicated drugs, according to guideline recommendations. However, since patients in lower NYHA classes need to receive fewer drugs than patients in higher NYHA classes, it is 'easier' to attain the score 'optimal' for the patients in lower classes, who in general indicate better QOL.

In addition, research suggested that one of the key elements to success in disease management programs seems to be an emphasis on patient education and self-management [19,21]. These elements help to give patients a sense of control over their condition and their ability to prevent deterioration, and therefore may strongly influence QOL [36]. Several trials showed better adherence to self-management strategies and improved QOL when patients received appropriate education [21]. However, a recent study found that participation in a quality improvement collaborative for heart failure was associated with better communication, knowledge, and lower health care use, but not with better QOL [37]. In bivariate analyses we found that patients in practices in which a) someone was responsible for self-management or b) in which materials were provided to practice members regarding the needs of HF patients, patients reported better HRQOL. However, both associations were rather limited in terms of clinical relevance and not statistically significant when we repeated the analysis adjusting for patient age, sex and NYHA class. Since NYHA class is consistently and closely associated to QOL [4], this presumably points to a selection effect; it is likely that a certain patient population (e.g. a higher number of patients in higher NYHA classes) is selected in certain practices (e.g. the ones who have a practice member who is responsible for self management). There is – however – a wide variety of programs and interventions that can be labelled 'disease management' [22], and therefore it is not always clear what particular element in such a 'comprehensive program' results in successful outcomes, which complicates the comparison of several studies with our results.

Some possible limitations of our study should be noted. Measuring lifestyle advice, we used self-reported data, as preventive and counselling activities have been found to be under recorded in EMRs [38]. However, we cannot rule out recall bias [36,39], which may have underestimated our measures. In addition, we did not measure whether the patient received advice from other caregivers than the GP, such as the cardiologist, which may have diluted a possible relationship between lifestyle advice received from the GP and patient HRQOL. Also, our measure of optimal pharmacological treatment did not take into account possible reasons to deviate from the suggestions in the guidelines such as contra indications and intolerance for drugs, multiple morbidities, or, simply, lack of robust evidence in case of chronic heart failure with preserved systolic function [40,41]. Future studies should preferably measure these factors – which may have caused underestimation of rates in our study [42,43]. Finally, we like to note that we cannot conclude that we showed causal linkages, as we used a cross-sectional design.

## Conclusion

This study is one of very few studies that explore the importance of structured care factors that may be related to HRQOL and high quality care in heart failure in a representative sample of primary care practices. The presence of structured care elements varied widely. However, only few associations were found between the structured care characteristics and optimal management and HRQOL. Better insight into the possible relevance of these factors is of importance to guide the design of future practice models in primary care that will contribute to high quality heart failure management, and – ultimately – higher HRQOL in patients. Future studies may benefit from more robust study designs, and combining quantitative and qualitative research methods [44] to disentangle what elements of disease management are effective and to gain more insights into possibly mediating or moderating factors. Also, more information on how to measure structured chronic care is needed [30], especially in primary care.

## Competing interests

The authors declare that they have no competing interests.

## Authors' contributions

MB, TW, RG, HS, RA, LN and MW designed the study. MB performed the data collection and data analyses, and all other authors contributed to interpreting the data. RA performed the multi-level logistic regression analyses. MB wrote the first draft, which was critically revised by all others. All authors have read and approved the final manuscript.

## Pre-publication history

The pre-publication history for this paper can be accessed here:


